# Natural Compounds in Glioblastoma Therapy: Preclinical Insights, Mechanistic Pathways, and Outlook

**DOI:** 10.3390/cancers13102317

**Published:** 2021-05-12

**Authors:** Kevin Zhai, Manaal Siddiqui, Basma Abdellatif, Alena Liskova, Peter Kubatka, Dietrich Büsselberg

**Affiliations:** 1 Department of Physiology and Biophysics, Weill Cornell Medicine-Qatar, Education City, Qatar Foundation, Doha P.O. Box 24144, Qatar; kez4003@qatar-med.cornell.edu (K.Z.); mas4002@qatar-med.cornell.edu (M.S.); bwa4001@qatar-med.cornell.edu (B.A.); 2 Department of Obstetrics and Gynecology, Jessenius Faculty of Medicine, Comenius University in Bratislava, 036 01 Martin, Slovakia; alenka.liskova@gmail.com; 3 Department of Medical Biology, Jessenius Faculty of Medicine, Comenius University in Bratislava, 036 01 Martin, Slovakia; peter.kubatka@uniba.sk

**Keywords:** glioblastoma, brain cancer, natural compounds, flavonoids, polyphenols, carotenoids, lignans, coumarins, steroids, tannins, terpenes, lifestyle medicine

## Abstract

**Simple Summary:**

Glioblastoma (GBM) is a tumor of the brain or spinal cord with poor clinical prognosis. Current interventions, such as chemotherapy and surgical tumor resection, are constrained by tumor invasion and cancer drug resistance. Dietary natural substances are therefore evaluated for their potential as agents in GBM treatment. Various substances found in fruits, vegetables, and other natural products restrict tumor growth and induce GBM cell death. These preclinical effects are promising but remain constrained by natural substances’ varying pharmacological properties. While many of the reviewed substances are available as over-the-counter supplements, their anti-GBM efficacy should be corroborated by clinical trials moving forward.

**Abstract:**

Glioblastoma (GBM) is an aggressive, often fatal astrocyte-derived tumor of the central nervous system. Conventional medical and surgical interventions have greatly improved survival rates; however, tumor heterogeneity, invasiveness, and chemotherapeutic resistance continue to pose clinical challenges. As such, dietary natural substances—an integral component of the lifestyle medicine approach to chronic diseases—are examined as potential chemotherapeutic agents. These heterogenous substances exert anti-GBM effects by upregulating apoptosis and autophagy, inducing cell cycle arrest, interfering with tumor metabolism, and inhibiting proliferation, neuroinflammation, chemoresistance, angiogenesis, and metastasis. Although these beneficial effects are promising, natural substances’ efficacy in GBM is constrained by their bioavailability and blood–brain barrier permeability; various chemical formulations are proposed to improve their pharmacological properties. Many of the reviewed substances are available as over-the-counter dietary supplements, underscoring their viability as lifestyle interventions. However, clinical trials remain necessary to substantiate the in vitro and in vivo properties of natural substances.

## 1. Glioblastoma: Occurrence, Mechanisms, Treatments, and Challenges

Glioblastoma (GBM) is a malignant tumor of the central nervous system (brain or spinal cord) that arises from astrocytes. It is the most common type of primary brain tumor, with occurrence rates of 3.19 cases per 100,000 patients in the United States, and 2.05 per 100,000 in the United Kingdom [1]. While the prognosis of GBM is often poor, two-year survival rates have improved in recent years, rising from 7% for cases diagnosed from 1993–1995 to 17% for cases diagnosed from 2005–2007 in the USA. Survival rates are also age-related: 39% of patients diagnosed between ages 20 and 44 survive, compared to only 1% of those diagnosed past age 80 [2].

While the efficacy of GBM treatment has improved, numerous challenges remain—especially concerning conventional therapeutic modalities. For instance, surgical tumor resection improves survival rates but is hindered by the extensive invasion and ill-defined tumor boundaries of GBM [3,4]. The efficacy of chemotherapeutic drugs may be reduced by the development of (multi-)drug resistance [5]. Moreover, extracranial metastasis—though rare—can greatly complicate treatment [6].

**Figure 1:**
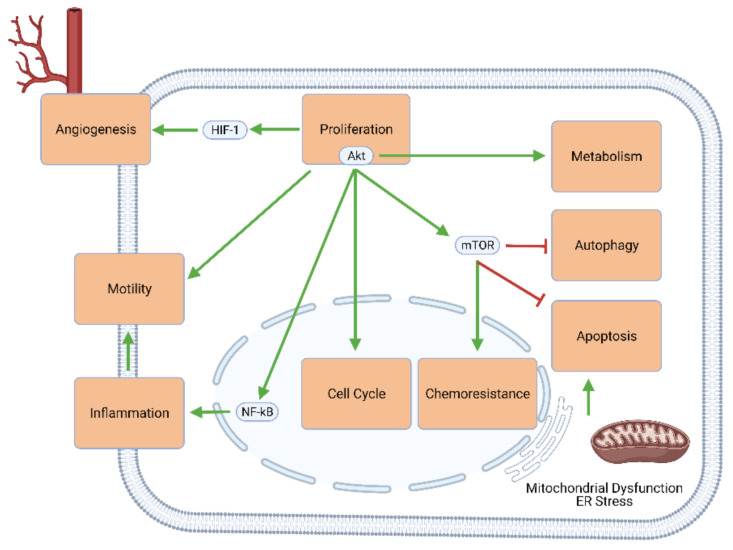
Intracellular signaling mechanisms involved in GBM development and progression. Elements of proliferative signaling pathways—especially Akt and mTOR—promote angiogenesis, motility and migratory potential, neuroinflammation, cell cycle progression, chemoresistance, and tumor metabolism, and concurrently inhibit GBM cell death through apoptosis and autophagy.

The challenges posed by GBM stem mainly from the genetic and molecular signaling pathways through which this type of tumor occurs. Genetic alterations in GBM include the amplification of the epidermal growth factor receptor (EGFR) and cyclin-dependent kinase (e.g., CDK4) genes, the deletion of the genes for cyclin-dependent kinase inhibitors (e.g., CDK2NA), and the silencing of the O-6-methylguanine-DNA methyltransferase (MGMT) gene [7]. These and other genetic changes upregulate cellular mechanisms that favor proliferation (e.g., through Akt/mTOR signaling), cell cycle progression, excessive and self-perpetuating inflammation, tumor metastasis, angiogenesis, metabolic changes (known as the Warburg effect), and chemoresistance. Simultaneously, the effectors of apoptosis and autophagy are largely downregulated or inhibited (Figure 1). As such, conventional oncologic therapies mostly aim to reverse this imbalance between growth and death by inhibiting proliferation and upregulating apoptosis.

The molecular complexity and difficulties posed by chronic diseases such as brain cancers have encouraged some clinicians to take a holistic approach to their treatment. Lifestyle medicine focuses on lifestyle factors (e.g., diet, physical activity, and the environment) and overall health maintenance to minimize risk factors associated with chronic diseases [8]. Dietary natural substances are an essential component of lifestyle medicine and can suppress cancer or overcome challenges associated with conventional therapies. Intake of these compounds may occur through the daily diet or over-the-counter supplements. While in vitro studies are promising, they are yet to be tangibly replicated in clinical trials.

## 2. Natural Compounds Modulating Glioblastoma

**Figure 2:**
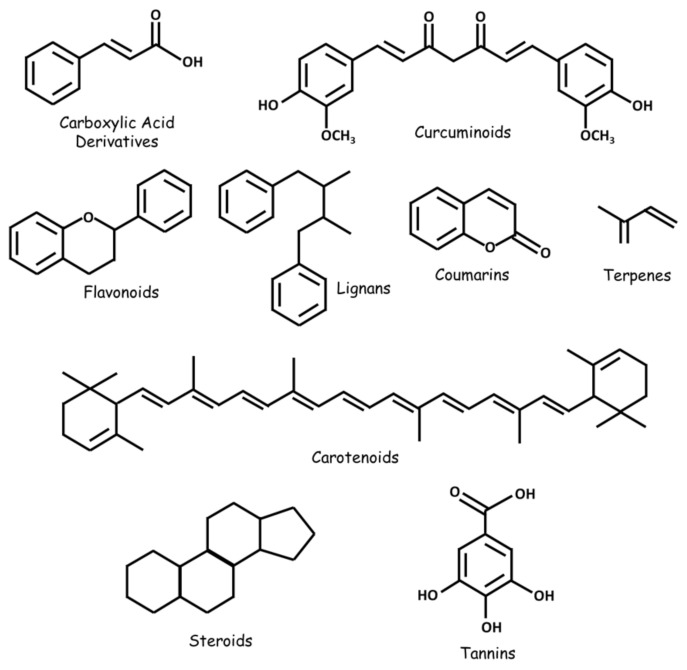
Some classes of natural substances with therapeutic potential in GBM.

**Figure 3:**
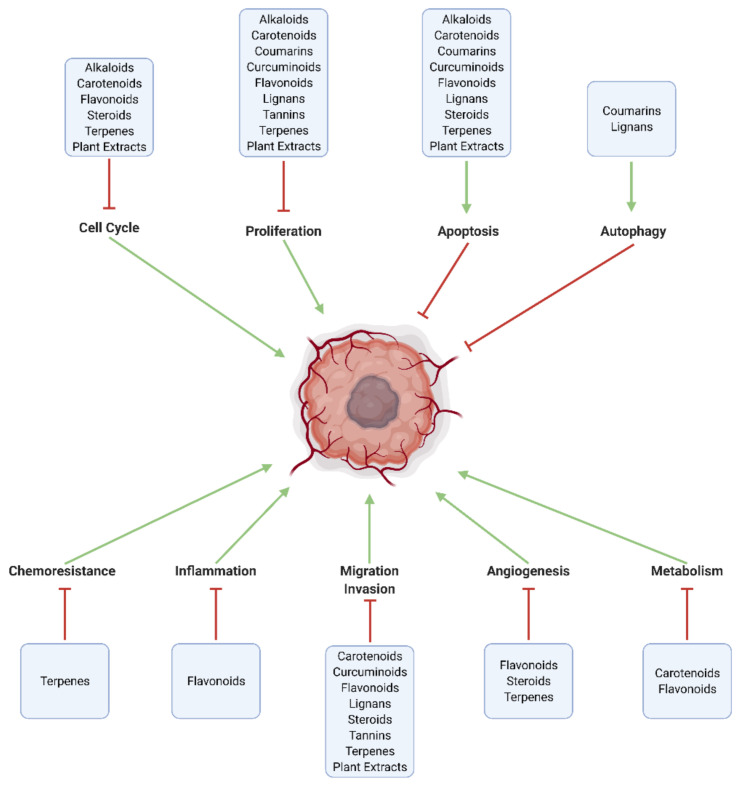
Major pathways modulated by natural substances in GBM. Effective chemotherapeutic substances increase cell death through apoptosis and autophagy, and inhibit intracellular mechanisms related to proliferation, cell cycle progression, tumor metabolism (Warburg effect), angiogenesis, invasion and metastasis, neuroinflammation, and chemoresistance.

Numerous natural substances—with established biological benefits—exert oncologic effects on GBM in vitro and/or in vivo. These include alkaloids, carboxylic acid derivatives, carotenoids, flavonoids, coumarins, curcuminoids, terpenes, lignans, natural steroids, tannins, and plant extracts (Figure 2 and Figure 3; Table 1).

### 2.1. Alkaloids

Alkaloids are structurally diverse, often basic natural compounds that contain at least one nitrogen atom. They also commonly contain oxygen atoms in organic rings. These compounds induce DNA damage, cell cycle arrest, ER stress, apoptosis, and autophagy, and concurrently inhibit angiogenesis and proliferation in tumor cells [9,10]. Moreover, alkaloids can overcome cancer drug resistance, as they inhibit cellular drug resistance pumps [10]. An alkaloid of interest in GBM therapy is berberine (BBR), a quaternary ammonium salt from barberry.

### 2.2. Carboxylic Acid Derivatives

Carboxylic acid derivatives are organic compounds with one or more carboxylic acid (COOH) functional groups. These organic acids have oncologic potential by modulating intracellular second messengers and suppressing DNA synthesis, transcriptional activity, and proliferation in tumor cells [11]. In recent years, cinnamic acid, a monocarboxylic acid from cinnamon, and ferulic acid, a cinnamic acid derivative from the giant fennel, have demonstrated anti-GBM efficacy in vitro.

### 2.3. Carotenoids

Carotenoids are red, orange, and yellow natural (often phytochemical) pigments. Two major classes exist within this family: (1) carotenes, which contain exclusively hydrogen and carbon atoms, and (2) xanthophylls, which contain oxygen atoms in addition to the hydrocarbon structure. Chemically, carotenoids are cyclic or acyclic tetraterpenoids with 40 carbon atoms—some of which are in conjugated double bond systems [12]. These pigments exert oncologic effects by upregulating the extrinsic and intrinsic apoptotic pathways in tumor cells [13]. They also disrupt tumor cell migration and invasion and thereby hinder metastasis [14]. Carotenoids discussed in this review include astaxanthin, a xanthophyll derived from chlorophyte; adonixanthin, a carotenone and derivative of astaxanthin; and crocetin, an apocarotenoid from saffron.

### 2.4. Flavonoids

Flavonoids are polyphenolic secondary metabolites of plants and occur in seven classes: anthocyanidins, flavones, flavanones, flavonols, flavan-3-ols, isoflavones, and chalcones. The flavonoid structural backbone is polyphenolic, with fifteen carbon atoms arranged in a three-ring structure. These compounds have well-characterized antitumor effects through the upregulation of apoptosis and disruption of migration, invasion, and metastasis [15,16]. Moreover, flavonoids modulate tumor cell glucose metabolism and downregulate the Warburg effect [17,18,19]. Flavonoids with anti-GBM potential include diosmin, a flavone glycoside from germander; epigallocatechin-3-gallate (EGCG), a catechin found mainly in green tea; matteucinol, a dihydroxyflavonone from naudin; naringin, a flavonone glycoside found in grapefruit and other citrus fruits; quercetin, a flavonol found in oak, onions, and kale; resveratrol, a stilbenoid found in grapes and red wine; rutin, a flavonol glycoside found in rue and citrus fruits; silymarin, an extract from milk thistle of which silibinin (a flavonolignan) is the major constituent; tectorigenin, a methylated isoflavone found in the leopard lily; chrysin, a dihydroxyflavone found in honey and propolis; galangin, a trihydroxylflavone that occurs in galangal; and xanthohumol, a chalcone (prenylated chalconoid) found in hops.

### 2.5. Coumarins

Coumarins are phenylpropanoid phytochemicals with one benzene ring and one heterocycle. These plant-derived compounds upregulate proapoptotic pathways, induce terminal differentiation, and reduce multi-drug resistance in cancerous cells [20]. Osthole, a coumarin found in Monnier’s snowparsley, and galbanic acid, a sesquiterpene coumarin abundant in *Ferula* species (*Apiaceae*), are coumarins of interest in GBM treatment.

### 2.6. Curcuminoids

Curcumin and its derivatives are (poly)phenolic plant secondary metabolites that comprise the curcuminoid family. Chemically, curcuminoids are diarylheptanoids, which have a seven carbon chain connecting two substituted aromatic rings. These polyphenols decrease proliferation and induce apoptosis, cell cycle arrest, and mitochondrial dysfunction in cancers of the nervous system [21]. They also notably modulate tumor angiogenesis and inflammation [22]. Curcumin, derived from the south Asian turmeric plant, exhibits inhibitory effects on GBM.

### 2.7. Terpenes

Unsaturated plant-derived hydrocarbons with the general chemical formula (C_5_H_8_)_n_ comprise the terpene family. These compounds are classified by the number of carbon atoms they contain; classes include monoterpenes (with 10 carbon atoms), sesquiterpenes (with 15), and diterpenes (with 20). Natural terpenes exert anticancer properties by inducing apoptosis and cell cycle arrest and suppressing tumor angiogenesis and metastasis [23,24]. Terpenes discussed in this review include AM01-06, sesquiterpene lactones from sunflower; betulinic acid, a triterpenoid from the white birch tree; cedrol, a sesquiterpene alcohol from the cypress and juniper trees; coronarin D, a diterpene from white ginger lily; eucalyptal A, a monoterpenoid from the southern blue gum tree; gossypol, a terpenoid aldehyde from cotton; paeoniflorin, a terpene glycoside from the Chinese peony; pisosterol, a triterpene from a fungus known as dead man’s foot; rupesin E, a monoterpenoid from Indian valerian; *Paris* saponin H, a triterpenoid saponin found in the Chong Lou plant; and tubeimoside-1, a triterpenoid saponin from the Chinese plant tu bei mu.

### 2.8. Lignans

Lignans are polyphenolic plant secondary metabolites that contain two benzene rings linked by carbon–carbon bonds. These natural polyphenols inhibit topoisomerases in tumor cells and thus interfere with DNA synthesis and proliferation [25]. Clinically, lignans decrease the risk of breast cancer [26]. Arctigenin, a lignan found in greater burdock, and magnolol, a biphenyl from the Houpu magnolia, have therapeutic potential in GBM.

### 2.9. Natural Steroids

Natural compounds containing the four-ring steroid nucleus—with 17 carbon atoms forming 3 cyclohexane (A, B, C) and 1 cyclopentane (D) rings—are classified as steroids. These biomolecules exert cytotoxic effects by inducing apoptosis and cell cycle arrest in tumor cells. Natural steroids can also target hormones, and as such may have anti-estrogen and anti-progestin properties [27]. Steroids of interest include withaferin A, a steroidal lactone from the Ashwa-gandha plant; N45, a steroidal saponin isolated from the Chinese medicinal plant nan chong lou; gamabufotalin, a steroidal lactone in the traditional Chinese medicine “ChanSu,” or toad skin extract; and diosgenin, a phytosteroid saponin found in Mediterranean fenugreek.

### 2.10. Tannins

Tannins are large, heavily hydroxylated polyphenols that can bind to (bio)macromolecules. They are classified by their base units: hydrolysable tannins (with gallic acid as the base unit), phlorotannins (phloroglucinol), and condensed tannins (flavan-3-ol). Tannins induce apoptosis and autophagy, inhibit proliferation, metastasis, and angiogenesis, and act synergistically with chemotherapeutics in cancerous cells [28]. Tannic acid, a hydrolysable tannin from oak, will be discussed in this review.

### 2.11. Crude and Purified Plant Extracts

Crude and purified plant extracts contain numerous chemical compounds with potential biological activities. Members of this highly heterogenous family with anti-GBM potential include BcH and BcS, water hyssop extracts sold as dietary supplements; aqueous and ethanol extracts from the shaggy ink cap (CW, CE70, and CE95), golden chanterelle (KW, KE70, and KE95), puffball (PW, PE70, and PE95), and saffron milk cap (RW, RE70, and RE95) mushrooms; CP, a chloroform partition from the johnnyberry plant; and PPE, an ethanol extract from Polish propolis.

## 3. Mechanistic Effects of Natural Compounds on Glioblastoma

### 3.1. Generalized Anti-Cancer Markers

Several generalizable effects can demonstrate the anti-GBM potential of natural compounds and highlight promising substances for further mechanistic studies (Table 2). Nearly all the substances discussed in this review decrease GBM cell viability in vitro. Cell viability assays are useful in (1) differentiating cytotoxic from biologically inert compounds and (2) identifying effective treatment concentrations to be used in further experiments. For example, decreased intracellular ATP is a marker of cell death; this effect was observed in GBM cells after treatment with curcumin, BBR, gossypol, and carnosine [29,30,31]. Several other substances, including xanthohumol and rupesin E, decreased cloning and colony formation—further indicators of cancer cell viability and malignancy—in GBM cultures.

The effects of some natural substances on GBM cells in culture are replicable in vivo, underscoring their therapeutic potential. Specific terpenes, carotenoids, flavonoids, and steroids inhibit tumor growth (measured through tumor area, perimeter, weight, and volume) in murine and rat xenograft models. Interestingly, the flavonoid matteucinol also reduces the area of GBM implants in fertilized chicken eggs. These effects may improve survival rates and times in tumor-bearing animals, as is the case for eucalyptal A, cedrol, and crocetin (see Table 2).

### 3.2. Proliferation, Apoptosis, and Autophagy

**Figure 4:**
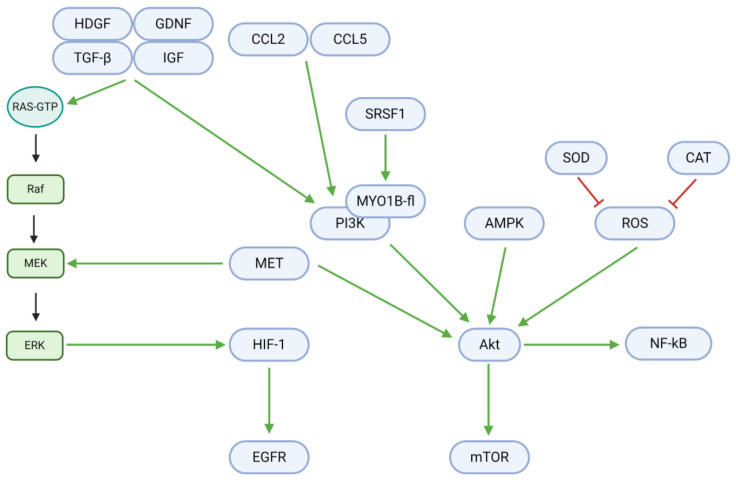
Intracellular mechanisms promoting proliferation in GBM. Growth factors, chemokine ligands, and other upstream signals upregulate the Ras/Raf/MEK/ERK and PI3K/Akt pathways. Downstream effectors, including HIF-1, EGFR, NF-κB, and mTOR, promote DNA synthesis, transcription, and tumor cell proliferation. Proliferative effectors notably engage in crosstalk with other signals in GBM, including those for angiogenesis (HIF-1), cell cycle progression (Akt), metabolism (Akt), motility (PI3K), apoptosis (Akt/mTOR), and autophagy (Akt/mTOR/Beclin-1).

Cell fate is regulated by a delicate balance between proliferation and death. In GBM and other tumor cells, growth factors, chemokine ligands, and other upstream signals mediate a shift towards excessive growth and proliferation (Figure 4; Table 3) [62]. Growth factors, including tumor growth factor βeta (TGF-β), insulin-like growth factor (IGF), hepatoma-derived growth factor (HDGF), and glial cell-derived neurotrophic factor (GDNF), are upregulated in GBM and contribute to downstream Ras/Raf/MEK/ERK and PI3K/Akt signaling. The upregulated chemokine (C-C motif) ligands 2 (CCL2) and 5 (CCL5) further contribute to the PI3K/Akt pathway. However, the flavonoids rutin and quercetin downregulate these proliferative signals in vitro and in vivo [63]. In the absence of natural inhibitory substances, the described growth factors and ligands bind to cell membrane receptors and activate Ras-GTP to begin the proliferative Ras/Raf/MEK/ERK pathway. In the first step, Ras-GTP activates Raf (a third degree MAPK, or MAP3K). Raf consequently activates the MAPK/ERK kinase (MEK; a second degree MAPK, or MAP2K)—an enzyme also activated by the MET proto-oncogene. MEK activates extracellular signal-regulated kinases (ERK) and their associated MAPKs in the third mechanistic step. Finally, ERK MAPKs upregulate hypoxia-inducible factor 1 αlpha (HIF-1α), whose downstream target is the epidermal growth factor receptor (EGFR). Osthole, a coumarin, may inhibit MEK activation in the second step through the downregulation of Raf [64]. TBMS1 may have a similar inhibitory function, as it downregulates MET [41]. Moreover, TBMS1, astaxanthin, and adonixanthin downregulate ERK/p-ERK to inhibit the final step of HIF-1α upregulation [35,41].

In addition to the Ras-GTP pathway, proliferation is also critically induced through Akt/mTOR and NF-κB signaling. Upstream of these targets, serine/arginine-rich splicing factor 1 (SRSF1) activates myosin 1B (MYO1B), which in turn upregulates the phosphoinositide-3-kinase (PI3K). PI3K, along with MET, adenosine monophosphate-activated protein kinase (AMPK), and reactive oxygen species (ROS), upregulates Akt, a central mediator of tumor cell proliferation. This step may be hindered by TBMS1, as it downregulates MET. Superoxide dismutase (SOD) and catalase (CAT) downregulate ROS levels and could therefore also inhibit Akt activation when upregulated by tannic acid and berberine [39,58]. Finally, Akt activity can be reduced through the downregulation of PI3K. Eucalyptal A downregulates PI3K by inhibiting SRSF1 and MYO1B, while curcumin, osthole, diosgenin, and berberine downregulate PI3K directly [29,32,54,64].

Downstream, Akt upregulates the mammalian target of rapamycin (mTOR) and nuclear factor κappa of activated B cells (NF-κB), which induce proliferation. However, arctigenin, curcumin, diosgenin, and berberine downregulate (p-)mTOR, while diosgenin downregulates NF-κB [29,54,61]. Moreover, galbanic acid exerts antiproliferative, anti-metastatic, and pro-apoptotic effects via PI3K/Akt/mTOR signaling, while N45, a natural steroidal saponin, upregulates apoptosis through ROS/PI3K/Akt signaling in TMZ-resistant GBM cells [65,66].

**Figure 5:**
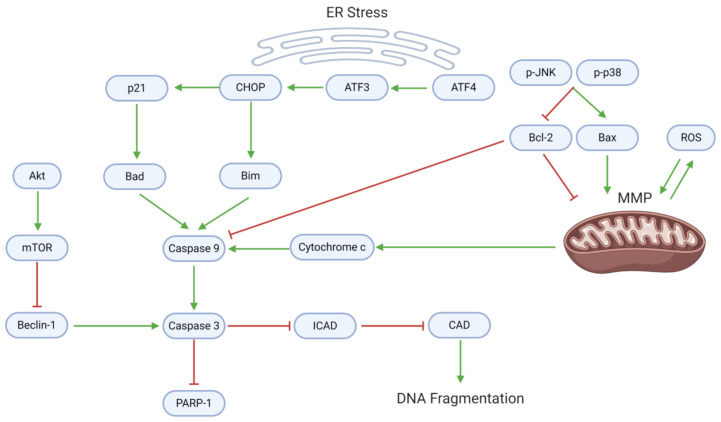
Proapoptotic mechanisms, which involve mitochondrial dysfunction, ER stress, and caspase activation, are suppressed in GBM. Dysregulation of mitochondrial homeostasis (often through oxidative imbalance) leads to the release of cytochrome c, a caspase activator. ER stress upregulates activating transcription factors; in turn, ATFs activate CHOP, p21, and proapoptotic proteins that enhance caspase activation. Active caspase 9 (along with Beclin-1) cleaves caspase 3, which enforces apoptosis and DNA fragmentation. In proliferating GBM cells, however, the anti-apoptotic protein Bcl-2 directly inhibits caspase 9, while mTOR inhibits Beclin-1.

Many natural substances’ anti-cancer properties arise from the activation of cell death through apoptosis and/or autophagy. Endoplasmic reticulum (ER) stress, mitochondrial dysfunction, and downstream caspase activity mediate the apoptotic death of GBM cells (Figure 5, Table 4). Withaferin A and EGCG upregulate activating transcription factor 4 (ATF4), an upstream effector of ER stress [40,42]. ATF4 targets ATF3, which consequently activates the C-homologous protein (CHOP). CHOP, which is also activated by withaferin A, upregulates p21 and the apoptotic proteins Bad and Bim [40].

In conjunction with ER stress, several mitochondrial mechanisms promote GBM cell apoptosis. Astaxanthin and adonixanthin upregulate (p-)p38 and associated MAPKs, which upregulate the proapoptotic Bax and downregulate the antiapoptotic Bcl-2 [35]. Alterations in the Bax:Bcl-2 ratio, mediated also by curcumin, berberine, pisosterol, and diosgenin, contribute to the depolarization of the mitochondrial membrane potential (MMP) [29,54]. Moreover, coronarin D, curcumin, and berberine upregulate intracellular ROS, further contributing to MMP depolarization [29,47,58]. MMP depolarization leads to cytochrome c release, as seen after treatment with curcumin or berberine [29].

Downstream, cytochrome c, Bad, and Bim promote the activation of caspase 9, which in turn activates caspase 3. A blockade of Akt/mTOR signaling mediated by arctigenin or osthole enhances the activity of Beclin-1, which supports caspase 3 activation [61,64]. Caspase 3 specifically blocks the inhibitor of caspase-activated DNAse (ICAD), allowing CAD to cause DNA fragmentation—an effect observed after diosgenin application [54]. Consequently, caspase 3 cleaves poly-ADP ribose polymerase 1 (PARP-1), activating apoptosis.

**Figure 6:**
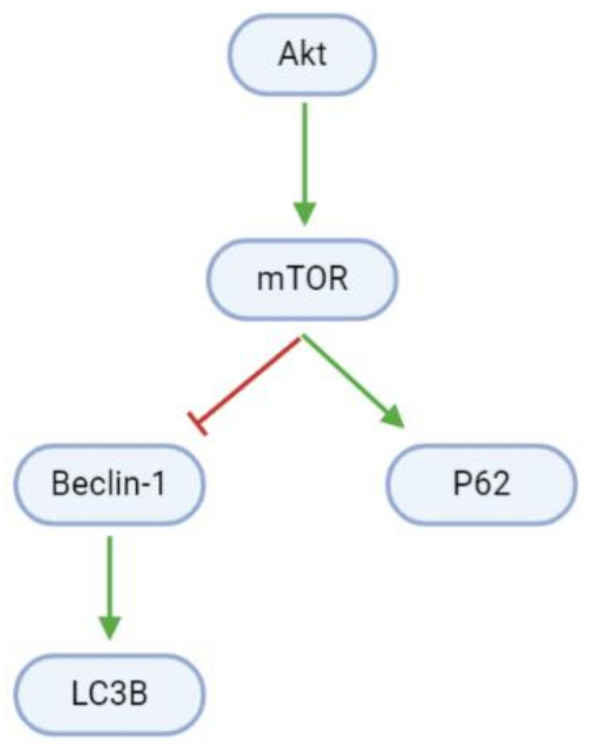
Pathways promoting cell death through autophagy are inhibited in GBM cells. mTOR inactivates the pro-autophagy Beclin-1 and upregulates the anti-autophagy P62.

Autophagy is blocked in proliferating GBM cells by Akt/mTOR signaling (Figure 6, Table 5). However, arctigenin and osthole upregulate Beclin-1 mRNA and protein levels. Beclin-1 interestingly has dual roles in apoptosis and autophagy, and upregulates light chain 3B (LC3B), which promotes autophagosome formation [61,64]. Moreover, arctigenin may increase autophagy through the upregulation of phosphorylated P62 (p-P62) [61].

### 3.3. Cell Cycle

**Figure 7:**
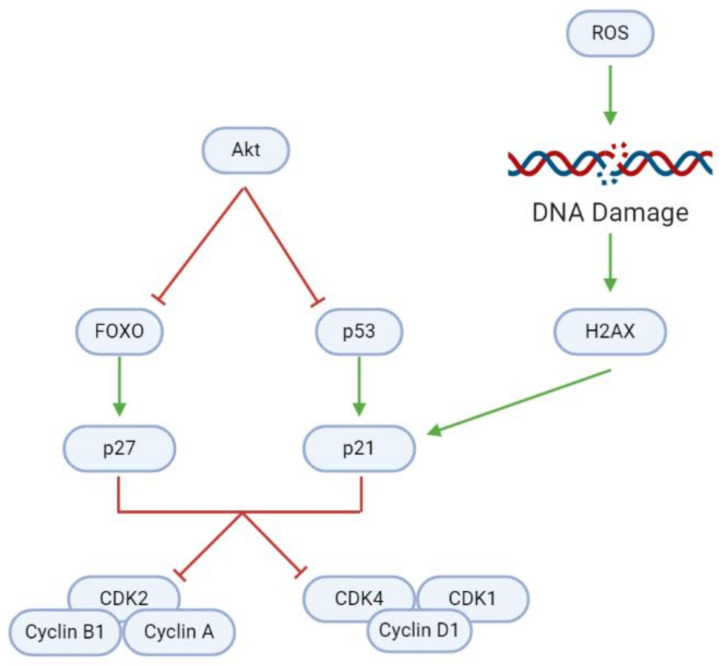
Inhibition of regulatory proteins allows for continuous cyclin/CDK activity and cell cycle progression in GBM cells. In healthy cells, FOXO and p53 can activate p27 and p21, respectively, and consequently induce cell cycle arrest to maintain homeostasis. DNA damage as a result of ROS accumulation is a key trigger for p21 activation. However, overactive Akt inhibits FOXO and p53, and therefore facilitates uncontrolled tumor cell growth and division.

In addition to modulating cell proliferation and death, natural substances may also affect tumorigenesis through the induction of cell cycle arrest (Figure 7, Table 6). Uncontrolled cell cycle progression due to the Akt-mediated inhibition of cyclin-dependent kinase (CDK) inhibitors (CDKNs) causes rapid GBM cell division. However, a blockade of Akt activates (1) forkhead homeobox O (FOXO), which in turn activates the CDKN p27; and (2) the p53 tumor suppressor, which activates the CDKN p21. Elevated intracellular ROS levels mediate further upregulation of p21. ROS damages DNA, upregulating H2A family member X (H2AX) and consequently p21, as evidenced after Coronarin D, CP, and McC1 treatment [36,47].

As CDKNs, p21 and p27 inhibit specific cyclin-CDK complexes that are necessary for cell cycle progression. Inhibition of CDK2, Cyclin A, and Cyclin B1, as seen after treatment with cedrol or TBMS1, leads to G2/M phase cell cycle arrest [33,41]. In contrast, *Paris* saponin H upregulates p21 and p27 and downregulates Cyclin D1, eventually causing G1 phase cell cycle arrest [69]. Likewise, the inhibition of CDK1, CDK4, and Cyclin D1 by Withaferin A, TBMS1, astaxanthin, adonixanthin, and cedrol prompts G0/G1 phase arrest [33,35,40,41].

### 3.4. Inflammation and Immune Cell Modulation

Neuroinflammation is an essential component of GBM tumorigenesis and interacts with various pro- and anticancer mechanisms (Table 7). Bispo da Silva et al. characterized rutin and quercetin’s pleiotropic effects on GBM-associated inflammation [63]. These flavonoids induce the chemotaxis and activation of microglia—resident macrophages in the nervous system—as evidenced by the immune cells’ adoption of amoeboid and multipolar morphologies. Moreover, rutin and quercetin promote microglial proliferation and migration to tumor sites, where they modulate cytokine levels and thereby affect the tumor inflammatory profile. For instance, rutin and quercetin treatment upregulates interleukins 1 (IL-1), 1-βeta (IL-1β), and 18 (IL-18)—pro-inflammatory cytokines of the IL-1 family. Chemokine (C-X3-C motif) ligand 1 (CX3CL1), which promotes microglial migration, is also activated. Concurrently, interleukins 4 (IL-4), 8 (IL-8), and 10 (IL-10), which have tumorigenic properties under certain circumstances, are downregulated.

Interestingly, the effects of natural compounds on interleukin 6 (IL-6) and tumor necrosis factor (TNF) vary between cell lines (see Table 7). Rutin and quercetin upregulate IL-6 at the mRNA level in C6 and TG1 (quercetin only) cells. However, along with CrataBL, they downregulate IL-6 at the mRNA level in U251 and TG1 (rutin only) cells and U251 xenografts in Wistar rats. They also downregulate IL-6 at the protein level in C6 and U87 cells. Similar pleiotropic effects are observed with TNF, which is upregulated at the mRNA and protein levels in U251, C6, and TG1 cells, but downregulated at the mRNA level in U251-Wistar rat xenograft models. These varying data underscore the need for further investigation into the immuno-modulatory properties of natural substances in GBM.

### 3.5. Migration, Invasion, and Metastasis

**Figure 8:**
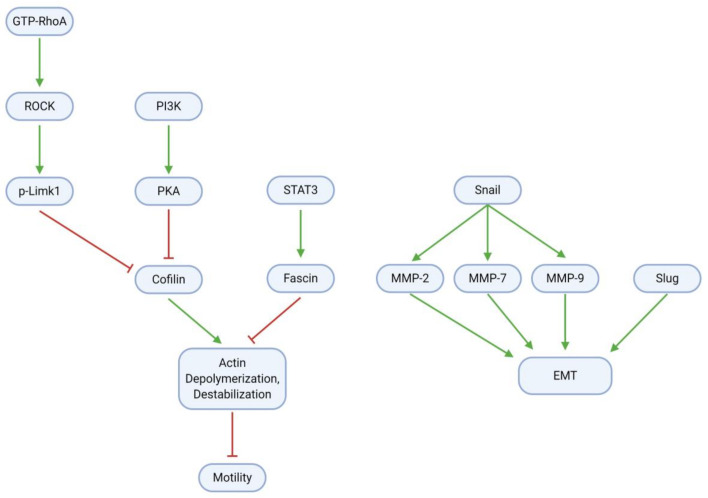
GBM cells gain migration and invasion abilities through EMT and modulation of the cytoskeletal actin framework. Regularization of actin filaments by STAT3/Fascin enhances cell motility; this process is reversible by Cofilin, which in tumor cells is inhibited by RhoA/ROCK/Limk and PI3K/PKA signaling. Upregulation of Snail, Slug, and MMPs further increases motility through EMT induction.

GBM cell migration, invasion, and metastasis are mainly mediated through the epithelial-mesenchymal transition (EMT) and modulation of the cytoskeletal actin framework (Figure 8; Table 8). To promote cell motility through actin, RhoA, a small GTPase, activates the Rho-associated protein kinase (ROCK); ROCK, in turn, activates the Lim kinase (Limk) through phosphorylation. Concurrently with RhoA/ROCK/Limk signaling, PI3K activates protein kinase A (PKA). Both Limk and PKA inhibit the activity of Cofilin (actin depolymerization factor), which ordinarily destabilizes cytoskeletal actin filaments and thereby impairs cell motility. Cofilin is active in the dephosphorylated form; as such, Limk and PKA may inhibit its activity through phosphorylation to produce p-Cofilin. Cofilin activity may be restored by paeoniflorin, which downregulates all components of the RhoA/ROCK/Limk pathway [53]. Eucalyptal A may also promote Cofilin activity, as it downregulates PKA [32]. Finally, the signal transducer and activator of transcription 3 (STAT3), a transcription factor commonly associated with inflammation, activates the actin bundling protein Fascin. Fascin acts antagonistically to Cofilin to stabilize the cytoskeleton and enhance cell motility; however, curcumin suppresses (p-)STAT3 and thereby downregulates Fascin activity [52].

Tumor cell adhesion and motility are further influenced by EMT, a process in which tumor cells become less adhesive and more migratory, and therefore more invasive. The Snail protein is upregulated in glioblastoma and activates the matrix metalloproteinases (MMP) 2, 7, and 9, which together with Slug contribute to the EMT. Several natural compounds have anti-EMT properties in GBM. These include TBMS1 and galangin, which directly downregulate Snail (and therefore the MMPs) and Slug [41,70]. Astaxanthin, adonixanthin, and diosgenin also downregulate MMPs; however, it remains unclear whether these effects are Snail-dependent [35]. Moreover, magnolol suppresses GBM cell migration by regulating focal adhesions and N-cadherin, while gamabufotalin demonstrates antimetastatic effects by downregulating urokinase plasminogen activator (uPA) and carbonic anhydrase 9 (CA9) and upregulating tissue inhibitor of metalloproteinases 3 (TIMP-3) [71,72].

**Table 8:** Natural substances decrease GBM cell migration and invasion by downregulating EMT modulators (Snail, Slug, and MMPs), Cofilin inhibitors (RhoA/ROCK/Limk and PKA), and actin polymerizers (STAT3/Fascin).

Effect	Substance	Cell Line	Source
Reduces cell migration	Eucalyptal A	U87MG, LN229	[32]
	Astaxanthin	GL261, U251MG	[35]
	Adonixanthin	GL261, U251MG	[35]
	Arctigenin	U87MG, T98G	[61]
	Crocetin	U87, U251	[34]
	CP	GAMG	[36]
	McC1	U251, GAMG	[36]
	Tannic Acid	C6	[39]
	TBMS1	U87, LN229	[41]
	Curcumin	U87	[52]
	Paeoniflorin	U251, T98G	[53]
	Diosgenin	C6, T98G	[54]
	Rutin	C6	[63]
	Magnolol	LN229, U87MG	[71]
	Gamabufotalin	U87	[72]
	Quercetin	C6	[63]
Reduces cell invasion	Eucalyptal A	U87MG, LN229	[32]
	Arctigenin	U87MG, T98G	[61]
	McC1	GAMG, U251	[36]
	CrataBL	U87	[49]
	Curcumin	U87	[52]
	Paeoniflorin	U251, T98G	[53]
	Diosgenin	C6, T98G	[54]
Downregulates MMP-2 (protein)	Astaxanthin	GL261	[35]
	Adonixanthin	GL261	[35]
	Arctigenin	U87MG	[61]
	TBMS1	U87, LN229	[41]
	Diosgenin	T98G	[54]
Downregulates MMP-7 (protein)	TBMS1	U87, LN229	[41]
Downregulates MMP-9 (protein)	Arctigenin	U87MG	[61]
	Diosgenin	C6	[54]
Downregulates p-PKA 1/2/3 (prot.)	Eucalyptal A	U87MG, LN229	[32]
Downregulates p-Cofilin (protein)	Eucalyptal A	U87MG, LN229	[32]
Downregulates fibronectin (protein)	Adonixanthin	GL261	[35]
Downregulates laminin (protein)	CrataBL	U87	[49]
Downregulates Snail (protein)	TBMS1	U87, LN229	[41]
	Galangin	U87, U251	[70]
Downregulates Snail (mRNA)	Galangin	U87, U251	[70]
Downregulates Slug (protein)	TBMS1	U87, LN229	[41]
Downregulates Fascin (protein)	Curcumin	U87	[52]
Reduces actin filament number	Paeoniflorin	T98G, U251	[53]
Downregulates GTP-RhoA (protein)	Paeoniflorin	T98G, U251	[53]
Downregulates ROCK (protein)	Paeoniflorin	T98G, U251	[53]
Downregulates (p-)Limk1 (protein)	Paeoniflorin	T98G, U251	[53]

### 3.6. Angiogenesis

Angiogenesis—the development of active blood vessels in and around tumor sites—is a key element of GBM progression (Table 9). Vascular endothelial growth factor (VEGF) mediates this process; it is upregulated by HIF-1 and downregulated by A disintegrin and metalloproteinase with thrombospondin motifs 1 (ADAMTS1). As such, substances such as *Paris* saponin H that inhibit HIF-1 will consequently downregulate VEGF [69]. The sesquiterpene lactone AM04 upregulates ADAMTS1 and thereby downregulates VEGF in U87 and T98G cells [44].

Reduced VEGF activity decreases tumor neovascularization; importantly, this is observable in vivo. Treatment of U87 xenografts in athymic mice with the flavonoid naringin downregulates tumor hemoglobin and the angiogenic markers cluster of differentiation 31 (CD31) and 105 (CD105) [37]. Moreover, matteucinol decreases the angiogenic area and the number of blood vessel junctions in a U251 xenograft-fertilized chicken egg model [36]. These in vivo effects demonstrate the potential applicability of specific natural substances as angiogenic modulators that inhibit GBM.

### 3.7. Metabolism

**Figure 9:**
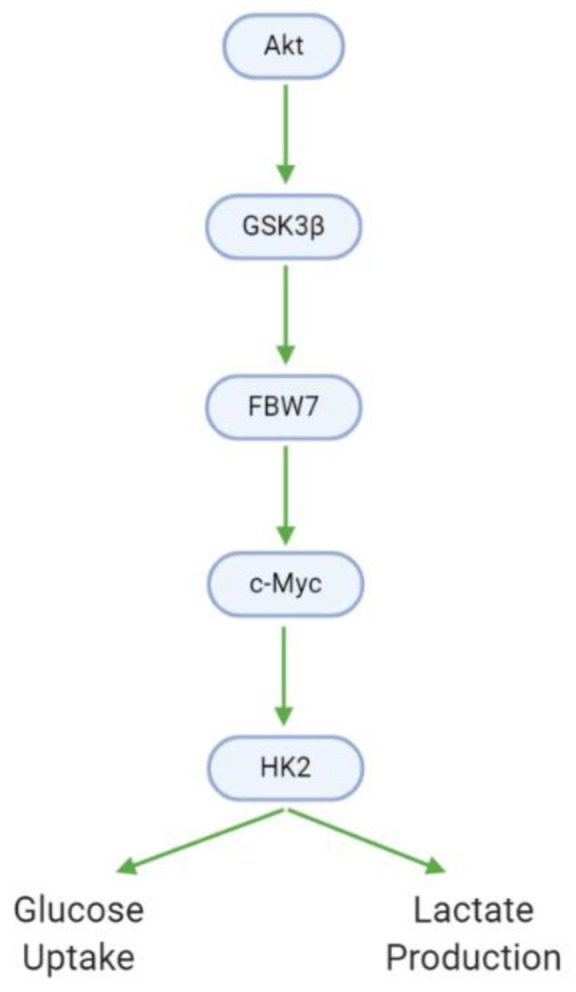
GBM cells utilize altered metabolic processes (Warburg effect) characterized by increased glucose uptake and lactate generation. Akt, via GSK3β, mediates the transition between the healthy and Warburg phenotypes.

Cancer cells exhibit modified metabolic processes that meet the extensive energy demands of growth, proliferation, and metastasis—a phenomenon known as the Warburg effect [73]. In GBM cells, Akt promotes glucose metabolism through the upregulation of glycogen synthase kinase 3 βeta (GSK3β). GSK3β, in turn, upregulates F-box and WD-40 domain-containing protein 7 (FBW7) and c-Myc, leading to the activation of hexokinase 2 (HK2). HK2 is a major metabolic enzyme that contributes to the aerobic glycolysis observed in tumor cells by increasing glucose uptake and lactate production (Figure 9, Table 10). Xanthohumol downregulates GSK3β, and as such decreases downstream HK2 activity, glucose consumption, and lactate production [38]. In contrast, carnosine upregulates pyruvate dehydrogenase kinase 4 (PDK4), which downregulates metabolism, while crocetin downregulates fatty acid synthase (FASN), which catalyzes metabolically relevant fatty acid synthesis [31,34].

### 3.8. Chemoresistance

The effects of natural substances on GBM chemoresistance remain largely uncharacterized in the recent literature. Chang et al. reported that cedrol downregulates O6-alkylguanine DNA alkyltransferase (MGMT) at the protein level in DBTRG-05MG and RG2 cells [33]. MGMT, a DNA repair protein, confers resistance to alkylating agents (e.g., temozolomide) by reversing guanine alkylation [74]. Studies from 2014 moreover indicate that pine needle extract, chrysin, and quercetin sensitize GBM cells to TMZ [75,76]. At any rate, further data are necessary to substantiate the potential of natural substances in overcoming GBM drug resistance.

## 4. Synergistic and Combinatory Effects (Multiple Natural Compounds)

While the actions of natural substances in conjunction with anticancer drugs are widely investigated, few studies have demonstrated the synergistic effects of concurrently applied natural substances in GBM (Table 11). One such study by Moskwa and colleagues demonstrates that Polish propolis (PPE) and BcH together decrease viability and DNA synthesis (a marker of proliferation) in T98G, LN-18, and U87 cells [57]. These findings demonstrate synergistic cytotoxic and anti-proliferative activities.

Another study by Maiti et al. shows that a combinatory treatment of solid lipid curcumin particles (SLCP) and BBR induces apoptosis and decreases proliferation and viability in U87 and U251 cells [29]. Synergistic apoptotic effects are mediated through the upregulation of ROS, Bax, cytochrome c, and caspases, while a blockade of PI3K/Akt/mTOR signaling decreases proliferation.

## 5. Challenges and Considerations in the Use of Natural Substances for GBM Treatment

### 5.1. Bioavailabilty and BBB Permeability

Bioavailability, metabolism, and blood–brain barrier (BBB) permeability are key factors in assessing natural substances’ clinical viability in GBM treatment. These properties vary widely between substances, and in many cases must be overcome to achieve sufficient in vivo and clinical concentrations. Oral bioavailability, a measure of a compound’s ability to reach the systemic circulation after ingestion, is lacking in several substances discussed in this review. BBR, for instance, has low oral bioavailability due to its poor absorption and rapid first-pass metabolism [77]. Osthole and curcumin are similarly constrained [21,78,79]. Furthermore, the flavonoids quercetin, naringin, and EGCG exhibit low oral bioavailability due to metabolic alterations and their high molecular weights [80]. Finally, the bioavailability of carotenoids varies with their structures; xanthophylls (which are more lipid-soluble) exhibit greater absorption than carotenes (which are purely hydrocarbon) [81]. In this light, the oral dosages necessary to replicate in vitro concentrations may be high and vary widely between substances.

Beyond bioavailability, BBB permeability is another consideration in designing brain-targeting therapies. Effective anti-GBM drugs must cross the BBB—a specialized endothelial cell layer that largely prevents passive diffusion between the brain and cranial blood vessels. Interestingly, BBR and coumarins, which have low oral bioavailability, are well absorbed through the BBB [82,83]. The BBB is furthermore permeable to xanthophylls (as evidenced by their cranial and retinal distribution), lipophilic flavonoids such as naringin and quercetin, and arctigenin [84,85,86]. In contrast, the flavonoid diosgenin does not appreciably cross the rat BBB after the administration of general yam extract [87]. Similarly, orally administered paeoniflorin cannot pass through the murine BBB [88].

While the bioavailability and BBB permeability of some natural compounds are independently corroborated, current preclinical research on GBM remains limited by poor modeling of physiological conditions and the BBB. Assessment of GBM cells in culture with high concentrations of natural products provides valuable mechanistic insights but does not reflect physiological realities. Several reviewed trials included animal models; however, some of these studies utilized heterotopic rather than orthotopic xenografts. Heterotopic—including subcutaneous—xenografts have limited utility, as they do not model the BBB. Orthotopic implants, in contrast, accurately model both the BBB and the in situ heterogeneity of brain tumors. Trials on orthotopic animal models therefore constitute an initial step in assessing natural compounds’ clinical viability. Of the reviewed substances, astaxanthin, adonixanthin, crocetin, eucalyptal A, tannic acid, rutin, and quercetin exerted appreciable anti-GBM effects in murine orthotopic models [32,34,35,39,63]. As such, these compounds are promising with regard to in situ bioavailability and BBB permeability.

### 5.2. Selectivity of Natural Compounds for GBM

In addition to favorable chemical and pharmacological properties, the selectivity of natural compounds for GBM cells is a key consideration for current preclinical and future clinical research. Effective chemotherapeutic agents should target cancerous cells while minimizing damage to healthy cells such as astrocytes. To this end, withaferin A, tannic acid, matteucinol, diosmin, cedrol, and rupesin E have been evaluated for GBM selectivity in vitro.

Recent preclinical studies reveal varying levels of GBM selectivity between compounds and cell lines. Tannic acid did not alter the viability of normal rat astrocytes at concentrations up to 75 µM, while diosmin at up to 150 µM remained minimally cytotoxic toward human astrocytes [39,46]. In contrast, withaferin A, cedrol, and rupesin E exhibited dose-dependent cytotoxicity; these compounds inhibited only GBM cells at lower concentrations, but both GBM cells and astrocytes at higher concentrations. Withaferin A was nontoxic toward HA1800 astrocytes at concentrations of 1 and 3 µM, but displayed significant cytotoxicity at 10 µM [40]. Cedrol also demonstrated this pattern of selectivity, with an IC_50_ for CTX TNA2 rat astrocytes more than two-fold higher than its IC_50_ values for DBTRG-05MG and RG2 GBM cell lines [33]. Similarly, the IC_50_ values of rupesin E were significantly greater for human cerebellar astrocytes than glioma stem cells [45]. Finally, matteucinol’s selectivity was cell line-dependent, with seemingly paradoxical selectivity indices of 1.60 and 0.36 for GAMG and U251 cells, respectively, compared to human astrocytes [36]. In this light, tannic acid and diosmin in particular are promising compounds; nevertheless, the in vivo selectivity of natural compounds and the variables that affect it must be clarified prior to the commencement of clinical trials.

### 5.3. Delivery Mechanisms to Enhance Natural Compounds’ Anti-GBM Properties

It is important to note that bioavailability, BBB permeability, and GBM selectivity are necessary for effective therapeutic design; high bioavailability alone without BBB permeability (or vice versa) is insufficient, and non-selective compounds could cause detrimental side effects. As such, numerous formulations were developed to enhance these properties of natural substances. For instance, protein complexes, microemulsions, nanosuspensions, and nanoparticles increase the bioavailability, absorption, and brain uptake of curcumin [21]. In particular, dodecamer peptide-functionalized polydopamine-coated curcumin-loaded zein nanoparticles effectively cross the BBB and deliver curcumin to GBM cells, with high penetration into 3D tumor spheroids. Although this delivery platform has great potential, it requires further in vivo evaluation [89]. Diosgenin-olive oil suspensions, quercetin polylactide-co-glycolide nanoencapsulations, and transferrin-modified osthole liposomes exhibit improved BBB permeability over the natural substances alone [87,90,91]. Chitosan-coated lipid microparticles and ApoE3-conjugated solid lipid nanoparticles improve resveratrol’s brain delivery [92]. Moreover, BBB-permeable theranostic photonic nanoparticles constitute an option for optically tracked drug delivery and release. Indeed, encapsulated visible and/or near-infrared photonic molecules in ultrasmall micellar structures with curcumin as a therapeutic and photonic component crossed the BBB and accumulated near orthotopic GBM xenografts. The intracranial delivery and release of curcumin is furthermore traceable through fluorescent imaging [93]. Indeed, phototheranostic nanoplatforms represent a promising approach for brain tumor imaging and therapy [94,95]. In conclusion, in vivo examination of these formulations represents a significant step forward; however, further standardization and experimentation are necessary prior to the commencement of clinical trials.

## 6. Implications for Lifestyle Medicine

### 6.1. Lifestyle Approaches to (Brain) Cancer

Lifestyle medicine focuses on changes in everyday habits, such as nutritional intake, physical activity levels, and risky behaviors (e.g., smoking). Clinical studies notably revealed that diet is a key factor modulating brain cancer risk and progression. For instance, Hu and colleagues correlated increased consumption of fresh vegetables and fruits with reduced risks of brain cancer development [96,97]. Moreover, the high fat, low carbohydrate ketogenic diet interferes with GBM cell glucose metabolism and demonstrates clinical efficacy [98,99].

Intake of some of the natural substances discussed in this review may be increased through simple dietary adjustments. In particular, grapefruit (naringin), grapes (rutin), green tea (EGCG), and cinnamon (cinnamic acid) are routinely consumed in various regions of the world. Other substances, however, may have non-dietary sources and/or be hampered by low intrinsic bioavailability, absorption, and BBB permeability; dietary supplements may be appropriate in these cases.

### 6.2. Availability of Natural Substances as Supplements

Over-the-counter (OTC) supplements may be utilized in various cases according to practical constraints and patient preferences. The Dietary Supplement Label Database (DSLD; https://dsld.od.nih.gov/dsld/ accessed on 2 February 2021), maintained by the US National Institutes of Health, provides insights into the availability of various natural compounds as supplements. Based on DSLD data, a number of the reviewed compounds are available in OTC supplements: the alkaloid BBR; the carboxylic acid derivatives cinnamic acid and ferulic acid; the carotenoids astaxanthin and crocetin; the coumarin osthole; curcumin; the flavonoids diosmin, EGCG, naringin, quercetin, resveratrol, chrysin, and rutin; the lignan magnolol; the natural steroids withaferin A and diosgenin; and the terpenes betulinic acid and paeoniflorin. Many of these supplements exert positive physiological effects; in particular, astaxanthin and curcumin positively influence the central nervous system [100,101,102,103,104,105,106,107,108,109,110,111,112].

### 6.3. Promising Natural Compounds and the Path to Clinical Trials

While a wide variety of natural compounds exhibit anti-GBM effects in vitro and in vivo, clinical trials are required to demonstrate their safety and efficacy. Candidate substances should be available on the market as OTC supplements, and demonstrate appreciable bioavailability, BBB permeability, and in situ selectivity for GBM. In this regard, rutin and quercetin hold promise as neuroinflammatory modulators in GBM: both flavonoids are available as OTC supplements, and their efficacy in murine orthotopic models demonstrates their bioavailability and BBB permeability [63]. The carotenoids astaxanthin and crocetin meet the same criteria [34,35]. Tannic acid is also promising, as it exhibits efficacy in orthotopic models as well as selectivity for GBM cells and minimal toxicity to astrocytes [39].

As these (and other) promising compounds progress toward clinical trials, the heterogeneity of brain tumors necessitates the cross-validation of such compounds’ mechanistic effects and selectivity between GBM cell lines. Physiological properties, such as bioavailability and BBB permeability, should be further clarified through preclinical studies with orthotopic tumor models and oral administration. If necessary, novel drug delivery systems can be designed to enhance these properties; however, formulations such as nanoparticles require standardization and safety evaluation prior to the commencement of clinical trials.

Finally, the potential for toxicity or adverse interactions between natural compounds and conventional drugs must be assessed and minimized. With regard to toxicity, four of the five promising compounds highlighted earlier in this section—rutin, quercetin, astaxanthin, and crocetin—are clinically safe [113,114,115,116]. Fewer studies concerning the fifth compound, tannic acid, are available; however, some of these trials reveal possible hepatotoxic and mutagenic effects [117]. Beyond toxicological studies, candidate natural compounds should be extensively trialed in vitro and in vivo with contemporary anti-GBM chemotherapeutics, such as TMZ, vincristine, carboplatin, etoposide, and irinotecan. Some recent studies revealed potentially detrimental drug–drug interactions. One pleiotropic flavonoid, quercetin, interacts dose-dependently with etoposide in vitro and modulates its efflux and metabolism in vivo, increasing the chemotherapeutic’s bioavailability and plasma concentration and decreasing its clearance [118,119]. In contrast, St. John’s Wort (which contains quercetin) decreases plasma irinotecan levels [120]. Moreover, low-dose quercetin reduces brain concentrations of vincristine—an important consideration in GBM therapy [121]. However, the same flavonoid exerts protective effects against vincristine-induced peripheral neurotoxicity [122]. Quercetin’s glycoside, rutin, is also a promising flavonoid; however, minimal data are available on its interactions with anti-GBM drugs. Data on astaxanthin, crocetin, and tannic acid are likewise limited; however, astaxanthin demonstrated protective effects against toxicity induced by cyclophosphamide, a salvage chemotherapeutic sometimes used in GBM. Notably, astaxanthin alleviated DNA damage—at the molecular and chromosomal levels—as well as oxidative stress in vivo [123]. These varying results underscore the need for further studies to elucidate the nature and implications of interactions between natural compounds and anti-GBM drugs.

## 7. Conclusions and Outlook

Natural compounds are an integral component of lifestyle medicine approaches to chronic diseases such as GBM. Members of the alkaloid, carboxylic acid derivative, carotenoid, coumarin, curcuminoid, flavonoid, lignan, steroid, tannin, and terpene families exert chemotherapeutic effects on GBM in vitro and in vivo. As such, they increase tumor cell death by upregulating pathways for autophagy and apoptosis and inhibiting those for proliferation. The reviewed substances concurrently induce cell cycle arrest, stabilize the neuroinflammatory profile, interfere with the Warburg effect, and inhibit angiogenesis and metastasis.

Although the results of in vitro and animal studies are promising, they remain uncorroborated by clinical trials. Importantly, natural substances’ clinical and lifestyle medical viability remain constrained by their pharmacokinetic and pharmacodynamic properties. Effective GBM therapies require appreciable oral bioavailability, BBB permeability, and GBM selectivity; however, the reviewed substances are highly heterogeneous in this regard. Various formulations are proposed to improve their pharmacological properties but are not yet clinically validated.

Finally, in evaluating natural compounds as chemotherapeutic agents in lifestyle medicine, their OTC availability must be considered. Many alkaloids, carboxylic acid derivatives, carotenoids, coumarins, curcuminoids, flavonoids, and natural steroids with in vitro anti-GBM efficacy are available in dietary supplements, while some of the discussed lignans, tannins, and terpenes are not. Caution is necessary in regular supplementation with natural substances, as the potential for adverse effects and/or drug–drug interactions exists.

In closing, recent preclinical studies underscore the viability of natural substances as candidate agents in GBM therapy. Innovative biochemical formulations could improve their physiological properties, and clinical trials could substantiate their beneficial effects.

## Figures and Tables

**Table 1:** Classes and sources of natural substances with anti-GBM efficacy demonstrated in recent preclinical studies. Many of the listed compounds occur in multiple natural sources.

Substance	Class/Type	Primary Source(s)
Alkaloids		
Berberine	Quaternary Ammonium Salt	Barberry (*Berberis*)
Carboxylic Acid Derivatives		
Cinnamic Acid	Monocarboxylic Acid	Cinnamon (*Cinnamomum*)
Ferulic Acid	Hydroxycinnamic Acid	Giant fennel (*Ferula communis*)
Carotenoids		
Adonixanthin	Carotenone	Derivative of astaxanthin
Astaxanthin	Xanthophyll	Chlorophyte (*Haematococcus pluvialis*)
Crocetin	Apocarotenoid	Saffron (*Crocus sativus*)
Coumarins		
Galbanic Acid	Sesquiterpene Coumarin	Celery/carrot/parsley family (*Umbelliferae*)
Osthole	Coumarin	Monnier’s snowparsley (*Cnidium monnieri)*
Curcuminoids		
Curcumin	Curcumin	Turmeric (*Curcuma longa*)
Flavonoids		
Chrysin	Dihydroxyflavone	Blue passion flower (*Passiflora caerulea*)
Diosmin	Flavone Glycoside	Germander (*Teucrium gnaphalodes*)
EGCG	Catechin	Green tea (*Camellia sinensis*)
Galangin	Trihydroxyflavone	Galangal (*Alpinia officinarum*)
Matteucinol	Dihydroxyflavonone	Naudin (*Miconia chamissois*)
Naringin	Flavanone Glycoside	Grapefruit (*Citrus × paradisi*)
Quercetin	Flavonol	Oak (*Quercetus*)
Resveratrol	Stilbenoid	Grape (*Vitis*)
Rutin	Flavonol Glycoside	Rue (*Ruta graveolens*)
Silymarin (Silibinin)	Flavonolignan	Milk thistle (*Silybum marianum*)
Tectorigenin	Methylated Isoflavone	Leopard lily (*Iris domestica*)
Xanthohumol	Prenylated Chalconoid	Hops (*Humulus lupulus*)
Lignans		
Arctigenin	Lignan/Polyphenol	Greater burdock (*Arctium lappa*)
Magnolol	Biphenyl	Houpu magnolia (*Magnolia officinalis*)
Steroids		
Diosgenin	Phytosteroid Sapogenin	Fenugreek (*Trigonella foenum-graecum*)
Gamabufotalin	Steroidal Lactone	Toad (*Bufo*)
N45	Steroidal Saponin	Nan chong lou (*Paris vietnamensis*)
Withaferin A	Steroidal Lactone	Ashwa-gandha (*Withania somnifera*)
Tannins		
Tannic Acid	Hydrolysable Tannin	Oak (*Quercetus*)
Terpenes		
AM01-06	Sesquiterpene Lactone	Sunflower (*Eremanthus* spp.)
Betulinic Acid	Triterpenoid	White birch (*Betula pubescens*)
Cedrol	Sesquiterpene Alcohol	Cypress (*Cupressus*); Juniper (*Juniperus*)
Coronarin D	Diterpene	White ginger lily (*Hedychium coronarium*)
Eucalyptal A	Monoterpenoid	Southern blue gum (*Eucalyptus globulus*)
Gossypol	Terpenoid Aldehyde	Cotton (*Gossypium*)
Paeoniflorin	Terpene Glycoside	Chinese peony (*Paeonia lactiflora*)
*Paris* saponin H	Triterpenoid Saponin	Chong Lou (*Rhizoma paridis*)
Pisosterol	Triterpene	Dead man’s foot (*Pisolithus tinctorius*)
Rupesin E	Iridoid (Monoterpenoid)	Indian valerian (*Valeriana jatamansi*)
Tubeimoside-1	Triterpenoid Saponin	Tu bei mu (*Rhizoma bolbostemmae*)
Crude/Purified Plant Extracts		
BcH, BcS	Extract-Food Supplement	Water hyssop (*Bacopa monnieri*)
CE70, CE95	Ethanol Extract	Shaggy ink cap (*Coprinus comatus*)
CP	Chloroform Partition	Johnnyberry (*Miconia chamissois*)
CW	Aqueous Extract	Shaggy ink cap (*Coprinus comatus*)
KE70, KE95	Ethanol Extract	Golden chanterelle (*Cantherellus cibarius*)
KW	Aqueous Extract	Golden chanterelle (*Cantherellus cibarius*)
PE70, PE95	Ethanol Extract	Puffball (*Lycoperdon perlatum*)
PPE	Ethanol Extract	Polish propolis (bee glue)
PW	Aqueous Extract	Puffball (*Lycoperdon perlatum*)
RE70, RE95	Ethanol Extract	Saffron milk cap (*Lactarius delicious*)
RW	Aqueous Extract	Saffron milk cap (*Lactarius delicious*)
Other		
Carnosine	Dipeptide	Liebig’s meat extract
CrataBL	Protein: Lectin + Serine Protease Inhibitor	Beach block (*Crataeva tapia*)
GL-PP	Polysaccharide Peptide	Lingzhi (*Ganoderma lucidum*)

**Table 2:** Generalized downstream effects of natural compounds on GBM. Many of the reviewed substances exert measurable cytotoxic effects in vitro. Moreover, several substances reduce tumor size and improve survival in-animal models of GBM.

Effect	Substance	Cell Line	Source
Increases survival	Eucalyptal A	U87MG orthotopic implants, nude mice	[32]
	Cedrol	DBTRG-05MG subcutaneous xenografts, nude mice	[33]
	Crocetin	Luc-U251MG orthotopic implants, CD1 mice	[34]
Decreases tumor area/perimeter	Astaxanthin	GL261 orthotopic implants, C57BL/6J mice	[35]
	Adonixanthin	GL261 orthotopic implants, C57BL/6J mice	[35]
	McC1	U251 heterotopic xenograft, fertilized chicken eggs	[36]
Decreases tumor volume	Astaxanthin	GL261 orthotopic implants, C57BL/6J mice	[35]
	Adonixanthin	GL261 orthotopic implants, C57BL/6J mice	[35]
	Naringin	U87 subcutaneous xenograft, athymic mice	[37]
	Xanthohumol	U87, LN229	[38]
	Tannic Acid	C6 orthotopic implants, Wistar rats	[39]
	Withaferin A	U87 subcutaneous xenografts, nude mice	[40]
	TBMS1	U87 subcutaneous xenografts, NOD/SCID mice	[41]
Decreases tumor weight	Xanthohumol	U87, LN229	[38]
	TBMS1	U87 subcutaneous xenografts, nude mice	[41]
Increases cell death/dec. viability	EGCG	U251, MO59J	[42]
	Cinnamic Acid	LN-229	[43]
	Ferulic Acid	LN-229	[43]
	Astaxanthin	GL261, U251MG	[35]
	Adonixanthin	GL261, U251MG	[35]
	Cedrol	DBTRG-05MG, RG2	[33]
	AM02	U87MG, T98G	[44]
	AM04	U87MG, T98G	[44]
	AM05	U87MG, T98G	[44]
	AM06	U87MG, T98G	[44]
	Naringin	U87	[37]
	Xanthohumol	U87, T98G, LN229	[38]
	Rupesin E	GSC-3#, GSC-12#, GSC-18#	[45]
	Diosmin	U87, GBM02, GBM95	[46]
	Coronarin D	U251	[47]
	CP	GAMG, U251	[36]
	McC1	GAMG, U251	[36]
	SLCP	U87, U251	[29]
	BBR	U87, U251	[29]
	Tannic Acid	C6	[39]
	Withaferin A	U87, U251	[40]
	Betulinic Acid	U251, LN229	[48]
	TBMS1	U87, LN229	[41]
	Carnosine	U87, T98G	[31]
	CrataBL	U87	[49]
	Tectorigenin	GBM-8401, GBM-8901	[50]
	Resveratrol	U87	[51]
	Quercetin	U87	[51]
	Curcumin	U87	[52]
	Paeoniflorin	U251, T98G	[53]
	Diosgenin	C6, T98G	[54]
	CW	LN-18	[55]
	CE70	U87, LN-18	[55]
	CE95	U87, LN-18	[55]
	KW	U87, LN-18	[55]
	KE70	U87, LN-18	[55]
	KE95	U87, LN-18	[55]
	RW	U87, LN-18	[55]
	RE70	U87, LN-18	[55]
	RE95	U87, LN-18	[55]
	PW	U87, LN-18	[55]
	PE70	U87, LN-18	[55]
	PE95	U87, LN-18	[55]
	Silymarin	U118	[56]
	BcS	U87, T98G, LN-18	[57]
	BcH	U87, T98G, LN-19	[57]
	BBR	U87	[58]
	GL-PP	U251	[59]
	Pisosterol	U87, U343, AHOL1, 1231N1	[60]
Decreases colony formation	Xanthohumol	U87, T98G, LN229	[38]
	Rupesin E	GSC-3#, GSC-18#	[45]
	CP	GAMG, U251	[36]
	McC1	U251, GAMG	[36]
	Tannic Acid	C6	[39]
Decreases cloning	Arctigenin	U87MG, T98G	[61]
	AM01	U87MG, T98G	[44]
	AM02	U87MG, T98G	[44]
	AM03	U87MG, T98G	[44]
	AM04	U87MG, T98G	[44]
	AM05	U87MG, T98G	[44]
	AM06	U87MG, T98G	[44]
	TBMS1	U87, LN229	[41]
Decreases sphere formation	Gossypol	TS13-20, TS13-18	[30]
Decreases intracellular ATP	SLCP	U87, U251	[29]
	BBR	U87, U251	[29]
	Gossypol	Diff13-20	[30]
	Carnosine	U87, T98G	[31]
Upregulates p53 (mRNA)	Pisosterol	U87, U343, AHOL1, 1231N1	[60]
Upregulates p53 (protein)	BBR	U87, U251	[29]
	SLCP	U251	[29]
	Pisosterol	U87, U343, AHOL1, 1231N1	[60]

**Table 3:** Natural substances decrease proliferation in GBM by downregulating upstream growth factors and chemokine ligands, components of the Ras/Raf/MEK/ERK and PI3K/Akt pathways, and downstream effectors.

Effect	Substance	Cell Line	Source
Decreases proliferation/growth	Rutin	C6	[63]
	Quercetin	C6	[63]
	Eucalyptal A	U87MG, LN229	[32]
	Rupesin E	GSC-3#, GSC-18#	[45]
	Crocetin	U87, U138, U251, U373	[34]
	Coronarin D	U251	[47]
	SLCP	U87, U251	[29]
	BBR	U87, U251	[29]
	Tannic Acid	C6	[39]
	Gossypol	Diff13-20, Diff13-18	[30]
	Betulinic Acid	U251, LN229	[48]
	CrataBL	U87	[49]
	Galbanic Acid	U87	[65]
	N45	U87	[66]
	Pisosterol	U87, U343, AHOL1, 1231N1	[60]
Decreases DNA synthesis	CE95	U87	[55]
	CE70	U87, LN-18	[55]
	KW	U87, LN-18	[55]
	KE95	U87, LN-18	[55]
	KE70	U87, LN-18	[55]
	PW	U87	[55]
	PE70	U87	[55]
	RW	U87, LN-18	[55]
	PPE	U87, T98G, LN-18	[57]
	BcH	U87, T98G, LN-18	[57]
Downregulates SRSF1 (mRNA)	Eucalyptal A	U87MG, LN229	[32]
Downregulates SRSF1 (protein)	Eucalyptal A	U87MG, LN229	[32]
Downregulates MYO1B-fl (protein)	Eucalyptal A	U87MG, LN229	[32]
Downregulates p-PDK1 (protein)	Eucalyptal A	U87MG, LN229	[32]
Downregulates TGF (mRNA)	Rutin	U251 orthotopic implants, WR	[63]
	Quercetin	U251 orthotopic implants, WR	[63]
Downregulates TGF-β (mRNA)	Rutin	C6	[63]
	Quercetin	C6	[63]
Downregulates IGF (mRNA)	Rutin	C6, WR-U251 orthotopic implants	[63]
	Quercetin	C6, WR-U251 orthotopic implants	[63]
Downregulates CCL2 (mRNA)	Rutin	U251 orthotopic implants, WR	[63]
	Quercetin	U251 orthotopic implants, WR	[63]
Upregulates CCL5 (mRNA)	Rutin	C6, WR-U251 orthotopic implants	[63]
	Quercetin	C6, WR-U251 orthotopic implants	[63]
Downregulates HDGF (mRNA)	Rutin	C6, WR-U251 orthotopic implants	[63]
	Quercetin	C6, WR-U251 orthotopic implants	[63]
Downregulates GDNF (mRNA)	Rutin	C6, WR-U251 orthotopic implants	[63]
	Quercetin	U251 orthotopic implants, WR	[63]
Downregulates PI3K (protein)	SLCP	U87	[29]
	BBR	U87	[29]
	Diosgenin	C6	[54]
Downregulates (p-)PI3K (protein)	Osthole	MOGGCCM, T98	[64]
	SLCP	U87, U251	[29]
	BBR	U87, U251	[29]
Upregulates AMPK (protein)	Metformin	U87	[67]
Downregulates Akt (mRNA)	Arctigenin	U87MG	[61]
Downregulates Akt (protein)	Cedrol	RG2	[33]
	Metformin	U87, U251	[68]
	SLCP	U87	[29]
	BBR	U87	[29]
Downregulates p-Akt (mRNA)	Arctigenin	U87MG, T98G	[61]
Downregulates p-Akt (protein)	Eucalyptal A	U87MG, LN229	[32]
	Astaxanthin	GL261	[35]
	Adonixanthin	GL261	[35]
	Cedrol	DBTRG-05MG, RG2	[33]
	Arctigenin	U87MG, T98G	[61]
	Xanthohumol	U87	[38]
	CP	GAMG	[36]
	McC1	GAMG, U251	[36]
	SLCP	U87, U251	[29]
	BBR	U87, U251	[29]
	Diosgenin	C6	[54]
Downregulates mTOR (protein)	Metformin	U87	[67]
	SLCP	U87	[29]
	BBR	U87, U251	[29]
Downregulates p-mTOR (mRNA)	Arctigenin	U87MG, T98G	[61]
Downregulates p-mTOR (protein)	Arctigenin	U87MG, T98G	[61]
	SLCP	U87	[29]
	BBR	U87, U251	[29]
	Diosgenin	T98G	[54]
Downregulates Raf (protein)	Osthole	MOGGCCM, T98	[64]
Downregulates c-Myc	Eucalyptal A	U87MG, LN229	[32]
	Xanthohumol	U87, T98G, LN229	[38]
	SLCP	U87	[29]
	BBR	U87	[29]
Downregulates ROS	Astaxanthin	GL261	[35]
	Adonixanthin	GL261	[35]
	Tannic Acid	C6	[39]
Upregulates CAT activity	Tannic Acid	C6	[39]
	BBR	U87	[58]
Upregulates SOD activity	Tannic Acid	C6	[39]
	BBR	U87	[58]
Downregulates JNK (protein)	Cedrol	DBTRG-05MG, RG2	[33]
Downregulates p-JNK (protein)	Cedrol	RG2	[33]
Downregulates p-MEK (protein)	TBMS1	U87, LN229	[41]
Downregulates p-ERK (protein)	Astaxanthin	GL261	[35]
	Adonixanthin	GL261	[35]
	TBMS1	LN229	[41]
Downregulates p38 (protein)	Diosgenin	T98G	[54]
Upregulates p-p38 MAPK (protein)	Astaxanthin	GL261	[35]
	Adonixanthin	GL261	[35]
Downregulates HIF-1α activity	Metformin	U251	[68]
Downregulates NF-κB	Diosgenin	C6, T98G	[54]
Downregulates MET (protein)	TBMS1	U87, LN229	[41]

**Table 4:** Natural substances increase apoptotic cell death in GBM by downregulating apoptotic inhibitors and upregulating active caspases, which cleave PARP-1 and induce DNA fragmentation.

Effect	Substance	Cell Line	Source
Causes apoptosis	Arctigenin	U87MG, T98G	[61]
	Osthole	MOGGCCM, T98	[64]
	Xanthohumol	U87	[38]
	Rupesin E	GSC-3#, GSC-18#	[45]
	Diosmin	GBM02, GBM95	[46]
	SLCP	U87, U251	[29]
	BBR	U87, U251	[29]
	Gossypol	TS13-20, Diff13-20	[30]
	Withaferin A	U87, U251	[40]
	Tectorigenin	GBM-8401, GBM-8901	[50]
	Diosgenin	C6, T98G	[54]
	Pisosterol	U87, U343, AHOL1, 1231N1	[60]
Causes DNA fragmentation	SLCP	U87, U251	[29]
	BBR	U87, U251	[29]
Upregulates (c-)caspase 9 (protein)	Cedrol	RG2	[33]
	Coronarin D	U251	[47]
	CP	GAMG	[36]
	McC1	GAMG	[36]
	Withaferin A	U87, U251	[40]
Upregulates caspase 3 (mRNA)	Pisosterol	U87, U343, AHOL1, 1231N1	[60]
Upregulates (c-)caspase 3 (protein)	EGCG	MO59J, U251	[42]
	Cedrol	DBTRG-05MG, RG2	[33]
	Osthole	T98	[64]
	Xanthohumol	U87, T98G, LN229	[38]
	Rupesin E	GSC-3#, GSC-18#	[45]
	Crocetin	U87, U138, U251, U373	[34]
	Diosmin	GBM02, GBM95	[46]
	Coronarin D	U251	[47]
	CP	GAMG	[36]
	McC1	GAMG, U251	[36]
	SLCP	U87, U251	[29]
	BBR	U87, U251	[29]
	Withaferin A	U87, U251	[40]
	Betulinic Acid	U251, LN229	[48]
	Resveratrol	U87	[51]
	Quercetin	U87	[51]
	GL-PP	U251	[59]
	Pisosterol	U87, U343, AHOL1, 1231N1	[60]
Upregulates (c-)PARP (protein)	Cedrol	RG2	[33]
	Xanthohumol	U87	[38]
	Coronarin D	U251	[47]
	CP	U251	[36]
	McC1	GAMG, U251	[36]
	Gossypol	TS13-20, Diff13-20	[30]
	Withaferin A	U87, U251	[40]
Downregulates PARP-1 (protein)	Diosgenin	C6, T98G	[54]
Downregulates ICAD (protein)	Diosgenin	C6, T98G	[54]
Upregulates Bax (protein)	SLCP	U87, U251	[29]
	BBR	U87, U251	[29]
	Diosgenin	C6, T98G	[54]
Downregulates Bcl-2 (mRNA)	Pisosterol	U87, U343, AHOL1, 1231N1	[60]
Downregulates Bcl-2 (protein)	Diosgenin	C6, T98G	[54]
	Pisosterol	U87, U343, AHOL1, 1231N1	[60]
Upregulates Bad (protein)	Withaferin A	U87, U251	[40]
Upregulates Bim (protein)	Withaferin A	U87, U251	[40]
Depolarizes MMP	Coronarin D	U251	[47]
	CP	U251	[36]
	McC1	U251	[36]
	Gossypol	TS13-20	[30]
	Withaferin A	U87, U251	[40]
Upregulates ROS	Coronarin D	U251	[47]
	SLCP	U87, U251	[29]
	BBR	U87, U251	[29]
Upregulates cytochrome c (protein)	SLCP	U87, U251	[29]
	BBR	U87, U251	[29]
Upregulates GRP78 (mRNA)	Withaferin A	U87, U251	[40]
Upregulates GRP78 (protein)	EGCG	MO59J	[42]
Upregulates ATF4 (mRNA)	Withaferin A	U87, U251	[40]
Upregulates ATF4 (protein)	Withaferin A	U87, U251	[40]
	EGCG	U251	[42]
Upregulates ATF6 (mRNA)	Withaferin A	U251	[40]
Upregulates XBP1 (mRNA)	Withaferin A	U87, U251	[40]
Upregulates XBP1 (protein)	Withaferin A	U87, U251	[40]
Upregulates CHOP (mRNA)	Withaferin A	U87, U251	[40]
Upregulates CHOP (protein)	Withaferin A	U87, U251	[40]
Upregulates Bax (protein)	Cedrol	DBTRG-05MG	[33]

**Table 5:** Arctigenin and osthole promote autophagy by upregulating Beclin-1 and LC3B-II and downregulating P62.

Effect	Substance	Cell Line	Source
Causes autophagy	Osthole	MOGGCCM	[64]
Upregulates Beclin-1 (mRNA)	Arctigenin	U87MG, T98G	[61]
Upregulates Beclin-1 (protein)	Arctigenin	U87MG, T98G	[61]
	Osthole	MOGGCCM	[64]
Upregulates LC3B-II (mRNA)	Arctigenin	U87MG, T98G	[61]
Upregulates LC3B-II (protein)	Arctigenin	U87MG	[61]
Downregulates P62 (mRNA)	Arctigenin	U87MG, T98G	[61]
Downregulates P62 (protein)	Arctigenin	U87MG, T98G	[61]

**Table 6:** Natural substances induce cell cycle arrest in GBM by upregulating p53, p21, and p27, and inhibiting several cyclins and their associated CDKs.

Effect	Substance	Cell Line	Source
Causes G0/G1 phase cell cycle arrest	Cedrol	DBTRG-05MG, RG2	[33]
	Coronarin D	U251	[47]
	Tannic Acid	C6	[39]
	Tectorigenin	GBM-8401	[50]
	BBR	U87	[58]
	GL-PP	U251	[59]
Causes G2/M phase cell cycle arrest	Eucalyptal A	U87MG, LN229	[32]
	Withaferin A	U87, U251	[40]
	TBMS1	U87, LN229	[41]
	Pisosterol	U87, U343, AHOL1, 1231N1	[60]
Downregulates Cyclin D1 (protein)	Astaxanthin	GL261	[35]
	Adonixanthin	GL261	[35]
	Cedrol	DBTRG-05MG	[33]
Downregulates CDK1 (protein)	Withaferin A	U87, U251	[40]
	TBMS1	U87	[41]
Downregulates CDK2 (protein)	Cedrol	DBTRG-05MG, RG2	[33]
Downregulates CDK4 (protein)	Tectorigenin	GBM-8401	[50]
Downregulates Cyclin A (protein)	Cedrol	DBTRG-05MG, RG2	[33]
	TBMS1	U87, LN229	[41]
Downregulates Cyclin B1 (protein)	Cedrol	DBTRG-05MG, RG2	[33]
	TBMS1	U87, LN229	[41]
Upregulates (p-)H2AX (protein)	Coronarin D	U251	[47]
	CP	U251	[36]
	McC1	GAMG, U251	[36]
Downregulates (p-)RB (protein)	Tectorigenin	GBM-8401	[50]
Upregulates p21 (protein)	Coronarin D	U251	[47]
	*Paris* saponin H	U251	[69]
	Withaferin A	U87, U251	[40]
	Tectorigenin	GBM-8401	[50]
Upregulates p27 (protein)	Astaxanthin	GL261	[35]
	*Paris* saponin H	U251	[69]
	Adonixanthin	GL261	[35]
	AM05	T98G	[44]

**Table 7:** Rutin, quercetin, and CrataBL exert pleiotropic and sometimes cell line-dependent effects on neuroinflammation.

Effect	Substance	Cell Line	Source
Activates microglia	Rutin	C6	[63]
	Quercetin	C6	[63]
Upregulates IL-1 (mRNA)	Rutin	U251 orthotopic implants, WR	[63]
	Quercetin	U251 orthotopic implants, WR	[63]
Upregulates IL-1β (mRNA)	Rutin	C6	[63]
	Quercetin	C6	[63]
Downregulates IL-4 (mRNA)	Rutin	U251 orthotopic implants, WR	[63]
	Quercetin	U251 orthotopic implants, WR	[63]
Upregulates IL-6 (mRNA)	Rutin	C6	[63]
	Quercetin	C6, TG1	[63]
Downregulates IL-6 (mRNA)	Rutin	U251, TG1, WR-U251 orthotopic implants	[63]
	Quercetin	U251, WR-U251 orthotopic implants	[63]
Downregulates IL-6 (protein)	Rutin	C6	[63]
	CrataBL	U87	[49]
Downregulates IL-8 (protein)	CrataBL	U87	[49]
Downregulates IL-10 (mRNA)	Rutin	C6, U251, TG1, WR-U251 orthotopic implants	[63]
	Quercetin	C6, U251, TG1, WR-U251 orthotopic implants	[63]
Downregulates IL-10 (protein)	Rutin	C6	[63]
Upregulates IL-18 (mRNA)	Rutin	U251 orthotopic implants, WR	[63]
	Quercetin	U251 orthotopic implants, WR	[63]
Upregulates TNF (mRNA)	Rutin	U251, TG1	[63]
	Quercetin	U251	[63]
Downregulates TNF (mRNA)	Rutin	U251 orthotopic implants, WR	[63]
	Quercetin	U251 orthotopic implants, WR	[63]
Upregulates TNF (protein)	Rutin	C6	[63]
Upregulates TNF-α (mRNA)	Rutin	C6	[63]
	Quercetin	C6	[63]
Upregulates CX3CL1 (mRNA)	Rutin	C6, WR-U251 orthotopic implants	[63]
	Quercetin	C6	[63]
Downregulates (p-)STAT3 (protein)	Curcumin	U87	[52]

**Table 9:** Natural substances reduce angiogenesis and neovascularization primarily by downregulating VEGF.

Effect	Substance	Cell Line	Source
Decreases angiogenesis area	McC1	U251 heterotopic xenograft, fertilized chicken eggs	[36]
Decreases blood vessel junctions	McC1	U251 heterotopic xenograft, fertilized chicken eggs	[36]
Decreases tube formation	Diosgenin	C6, T98G	[54]
Upregulates ADAMTS1 (protein)	AM04	U87MG, T98G	[44]
Downregulates CD31 (mRNA)	Naringin	U87 subcutaneous xenograft, athymic mice	[37]
Downregulates CD105 (mRNA)	Naringin	U87 subcutaneous xenograft, athymic mice	[37]
Downregulates tumor hemoglobin	Naringin	U87 subcutaneous xenograft, athymic mice	[37]
Downregulates VEGF (protein)	Metformin	U251	[68]
	*Paris* saponin H	U251	[69]
	CrataBL	U87	[49]
	Diosgenin	C6	[54]

**Table 10:** Xanthohumol, carnosine, and crocetin interfere with key enzymes in GBM cell metabolism.

Effect	Substance	Cell Line(s)	Source
Downregulates HK2 (protein)	Xanthohumol	U87, T98G, LN229	[38]
Decreases glucose consumption	Xanthohumol	U87, T98G, LN229	[38]
Decreases lactate production	Xanthohumol	U87, T98G, LN229	[38]
Downregulates (p-)GSK3β (protein)	Xanthohumol	U87	[38]
Upregulates PDK4 (mRNA)	Carnosine	U87, T98G	[31]
Downregulates FASN (protein)	Crocetin	U87, U138, U251, U373	[34]

**Table 11:** Synergistic effects of natural substances on GBM. Concurrent administration of PPE and BcH enhances GBM cytotoxicity and reduces DNA synthesis, while curcumin and BBR together reduce proliferation and increase apoptotic cell death.

Effect	Cell Line	Subs. 1	Subs. 1 Conc.	Subs. 2	Subs. 2 Conc.	Source
Increases cell death/dec viability	U87	SLCP	20 µM	BBR	100 µM	[29]
	U251	SLCP	20 µM	BBR	100 µM	[29]
	T98G	PPE	30 µg/mL	BcH	5, 10, 25, 50, 100 µg/mL	[57]
	LN-18	PPE	30 µg/mL	BcH	50, 100 µg/mL	[57]
	U87	PPE	30 µg/mL	BcH	50, 100 µg/mL	[57]
Decreases proliferation	U87	SLCP	20 µM	BBR	100 µM	[29]
	U251	SLCP	20 µM	BBR	100 µM	[29]
Causes apoptosis	U87	SLCP	20 µM	BBR	100 µM	[29]
	U251	SLCP	20 µM	BBR	100 µM	[29]
Causes DNA fragmentation	U87	SLCP	20 µM	BBR	100 µM	[29]
	U251	SLCP	20 µM	BBR	100 µM	[29]
Decreases DNA synthesis	T98G	PPE	30 µg/mL	BcH	25, 50 µg/mL	[57]
	LN-18	PPE	30 µg/mL	BcH	25, 50 µg/mL	[57]
	U87	PPE	30 µg/mL	BcH	25, 50 µg/mL	[57]
Decreases intracellular ATP	U87	SLCP	20 µM	BBR	100 µM	[29]
	U251	SLCP	20 µM	BBR	100 µM	[29]
Upregulates ROS	U87	SLCP	20 µM	BBR	100 µM	[29]
	U251	SLCP	20 µM	BBR	100 µM	[29]
Upregulates Bax (protein)	U87	SLCP	20 µM	BBR	100 µM	[29]
	U251	SLCP	20 µM	BBR	100 µM	[29]
Upregulates cytochrome c (protein)	U87	SLCP	20 µM	BBR	100 µM	[29]
	U251	SLCP	20 µM	BBR	100 µM	[29]
Upregulates (c-)caspase 3	U87	SLCP	20 µM	BBR	100 µM	[29]
	U251	SLCP	20 µM	BBR	100 µM	[29]
Downregulates c-Myc (protein)	U87	SLCP	20 µM	BBR	100 µM	[29]
	U251	SLCP	20 µM	BBR	100 µM	[29]
Upregulates p53 (protein)	U87	SLCP	20 µM	BBR	100 µM	[29]
	U251	SLCP	20 µM	BBR	100 µM	[29]
Downregulates Akt (protein)	U87	SLCP	20 µM	BBR	100 µM	[29]
Downregulates (p-)Akt (protein)	U87	SLCP	20 µM	BBR	100 µM	[29]
	U251	SLCP	20 µM	BBR	100 µM	[29]
Downregulates PI3K (protein)	U87	SLCP	20 µM	BBR	100 µM	[29]
Downregulates (p-)PI3K (protein)	U87	SLCP	20 µM	BBR	100 µM	[29]
	U251	SLCP	20 µM	BBR	100 µM	[29]
Downregulates mTOR (protein)	U87	SLCP	20 µM	BBR	100 µM	[29]
	U251	SLCP	20 µM	BBR	100 µM	[29]
Downregulates (p-)mTOR (protein)	U87	SLCP	20 µM	BBR	100 µM	[29]
	U251	SLCP	20 µM	BBR	100 µM	[29]

## Abbreviations

ADAMTS1	A Disintegrin And Metalloproteinase with ThromboSpondin motifs 1
Akt	serine-threonine kinase Akt
AM01	4β,5-dihydro-15-deoxy-eremantholide
AM02	4β,5-dihydro-2′,3′-epoxy-15-deoxy-goyazensolide
AM03	4β,5-dihydro-1′,2′-epoxy-15-deoxy-eremantholide
AM04	goyazensolide
AM05	lychnofolide
AM06	15-deoxy-goyazenolide
AMPK	5’ Adenosine Monophosphate-activated Protein Kinase
APC	κN′,N’’-3-acetyloxy-BA-28-[2-(2-aminoethyl)aminoethyl]amide dichlorido platinum(II)
ATF4	Activating Transcription Factor 4
ATF6	Activating Transcription Factor 6
Bad	Bcl-2 associated death promoter
Bax	Bcl-2 associated x protein
BBR	BerBeRine
Bcl-2	B-cell lymphoma 2
c-Myc	MYC proto-oncogene
CA9	Carbonic Anhydrase 9
CAD	Caspase-Activated DNAse
CAT	CATalase
CCL2	C-C motif chemokine Ligand 2
CCL5	C-C motif chemokine Ligand 5
CD105	Cluster of Differentiation 105
CD31	Cluster of Differentiation 31
CDK2	Cyclin-Dependent Kinase 2
CDK4	Cyclin-Dependent Kinase 4
CHOP	C/EBP HOmologous Protein
CX3CL1	chemokine (C-X3-C motif) Ligand 1
DE9B	3-acetyloxy-BA-28-[2-(2-aminoethyl)aminoethyl]amide
DSLD	Dietary Supplement Label Database
ECAR	ExtraCellular Acidification Rate
EGFR	Epidermal Growth Factor Receptor
EMT	Epithelial-Mesenchymal Transition
ERK	Extracellular signal-Regulated Kinase
FASN	Fatty Acid SyNthase
FBW7	F-Box and WD repeat domain-containing 7
FPR1	Formyl Peptide Receptor 1
GBM	GlioBlastoMa
GDNF	Glial cell-Derived Neurotrophic Factor
GSC	Glioma Stem Cell
GSK3β	Glycogen Synthase Kinase 3 βeta
GTP-RhoA	Guanosine TriPhosphate-RhoA
H2AX	H2A histone family member X
HDAC1	Histone DeACetylase 1
HDAC3	Histone DeACetylase 3
HDGF	Hepatoma-Derived Growth Factor
HIF-1α	Hypoxia Inducible Factor-1 αlpha
HK2	HexoKinase 2
ICAD	Inhibitor of Caspase-Activated DNAse
IGF	Insulin-like Growth Factor
IL-1(β)	InterLeukin 1(βeta)
IL-18	InterLeukin 18
IL-4	InterLeukin 4
IL-6	InterLeukin 6
IL-10	InterLeukin 10
JNK	c-Jun N-terminal Kinase
LC3B-II	microtubule-associated proteins 1A/1B Light Chain 3B
MAPK	Mitogen Activated Protein Kinase
Mcl-1	Myeloid cell leukemia 1
MDR1	MultiDrug Resistance protein 1
MGMT	O^6^-MethlyGuanine-DNA-MethylTransferase
MMP-2	Matrix MetalloProteinase-2
MMP-9	Matrix MetalloProteinase-9
mTOR	mammalian Target Of Rapamycin
MYO1B	MYOsin 1B
NOS2	Nitric Oxide Synthase 2
Nox-4	NADPH oxidase 4
OCR	Oxygen Consumption Rate
OS	Overall Survival
OTC	Over-The-Counter
PAK 1/2/3	p21/Cdc42/Rac1-Activated Kinase 1/2/3
PARP	Poly (ADP-Ribose) Polymerase
PDK1	Pyruvate Dehydrogenase Kinase 1
PDK4	Pyruvate Dehydrogenase Kinase 4
PFS	Progression Free Survival
PI3K	PhosphoInositide 3-Kinase
PTGS2	ProsTaGlandin-endoperoxide Synthase 2
PTPN1	Protein Tyrosine Phosphatase Non-receptor type 1
Raf	Rapidly accelerated fibrosarcoma
ROCK	RhO-assoCiated protein Kinase
ROS	Reactive Oxygen Species
SLCP	Solid Lipid Curcumin Particles
SOD	SuperOxide Dismutase
SRSF1	Serine/arginine-Rich Splicing Factor 1
TBMS1	TuBeiMoSide-1
TGF(-β)	Tumor Growth Factor (βeta)
TIMP-3	Tissue Inhibitor of MetalloProteinases 3
TMZ	TeMoZolomide
TNF(-α)	Tumor Necrosis Factor (αlpha)
uPA	urokinase Plasminogen Activator
VEGF	Vascular Endothelial Growth Factor
XBP1	X-box Binding Protein 1
